# Supplementary material for: Development and Validation of a New Home Cooking Frequency Questionnaire: A Pilot Study
Source: Nutrients. 2022 Mar 8;14(6):1136. doi: 10.3390/nu14061136 (PMC8950242; doi:10.3390/nu14061136)
Supplement: Supplementary file 1 [file nutrients-14-01136-s001.zip › nutrients-1589567-supplementary.pdf]

SUPPLEMENTARY MATERIAL

Supplementary Material Section S1. Home cooking frequency questionnaire in Spanish (validated) and translate version (not validated) in English.

CUESTIONARIO DE FRECUENCIA DE ACTIVIDADES CULINARIAS EN EL HOGAR

Este cuestionario tiene como objetivo conocer sus hábitos culinarios, así como el modo de cocinar en casa los distintos grupos de alimentos. Todas las preguntas hacen referencia al término medio durante el último año.

1. ¿Se encarga usted de planificar los menús semanales de comidas y/o cenas en casa?
- ☐ Sí, soy el encargado de la planificación semanal de comidas y/o cenas
- ☐ No, pero sí planificamos semanalmente las comidas y/o cenas
- ☐ No planificamos semanalmente las comidas y/o cenas

2. ¿Se encarga o colabora usted con la compra semanal de su hogar? ☐ Sí ☐ No

3. ¿Cocina usted en casa? ☐ Sí ☐ No ***(pase a pregunta 8)***

4. ¿Qué porcentaje de los platos elaborados en su casa los cocina usted? ☐ <25% ☐ 25-49% ☐ 50-75% ☐ >75% ☐ 100%

5. ¿Para cuántas personas cocina incluyéndose a usted mismo? ☐ 1 ☐ 2 ☐ 3 ☐ 4 ☐ 5 ☐ 6 ☐ 7 ☐ 8 ó +

6. ¿Practica "batch cooking"? *(batch cooking: cocinar un día de la semana las comidas y/o cenas de varios días)* ☐ Sí ☐ No

Por favor, marque una respuesta para ambas preguntas

	DÍAS A LA SEMANA							HORAS A LA SEMANA		
	1 día	2 días	3 días	4 días	5 días	6 días	7 días	<3h	3-7h	>7h
**7. ¿Cuántos días y horas a la semana dedica a cocinar?** *(no incluye periodos de vacaciones, Navidad, Semana Santa, etc…)*	☐	☐	☐	☐	☐	☐	☐	☐	☐	☐
**8. ¿Cuántos días y horas a la semana dedica a cocinar en periodos estivales (verano, Navidad, Semana Santa, etc…)?**	☐	☐	☐	☐	☐	☐	☐	☐	☐	☐

Para cada una de las siguientes preguntas, marque el recuadro que indica la frecuencia **por término medio** durante el **año pasado**.

**9. ¿Con qué frecuencia usted…**	FRECUENCIA MEDIA DURANTE EL AÑO PASADO								
	NUNCA O CASI NUNCA	AL MES	A LA SEMANA						
		1-3	1	2	3	4	5	6	7
come comida cocinada en casa (incluye tupper, casa de un familiar, amigo/a)?	☐	☐	☐	☐	☐	☐	☐	☐	☐
cena comida cocinada en casa (incluye tupper, casa de un familiar, amigo/a)?	☐	☐	☐	☐	☐	☐	☐	☐	☐
come y/o cena usted acompañado/a?	☐	☐	☐	☐	☐	☐	☐	☐	☐
come en restaurantes o en comedor de empresa?	☐	☐	☐	☐	☐	☐	☐	☐	☐
cena en restaurantes o en comedor de empresa?	☐	☐	☐	☐	☐	☐	☐	☐	☐
compra comida casera para llevar de restaurantes o supermercados?	☐	☐	☐	☐	☐	☐	☐	☐	☐
consume alimentos precocinados en la comida (sopas instantáneas, pizzas…)?	☐	☐	☐	☐	☐	☐	☐	☐	☐
consume alimentos precocinados en la cena (sopas instantáneas, pizzas…)?	☐	☐	☐	☐	☐	☐	☐	☐	☐

A continuación, las preguntas se refieren a las técnicas culinarias que se emplean en su hogar para cocinar. Responda por favor, aunque usted no cocine. Para cada grupo de alimentos indique la frecuencia con la que se ha empleado cada técnica culinaria por término medio durante el último año. Cuando hablamos de cocinar nos referimos a cualquier tipo de procesamiento de alimentos frescos, congelados o embotados. No consideramos cocinar la manipulación de alimentos ya preparados o casi preparados como por ejemplo pizzas o lasañas preparadas, sopas o cremas de sobre, o arroz o pasta listos para calentar en el microondas, entre otros.

Se facilita una lista de las técnicas culinarias más frecuentes y su definición, que le puede ser útil para rellenar el cuestionario		**EJEMPLOS**
A la plancha	Cocción a alta temperatura y en medio seco (puede añadirse una gota de aceite)	*Pollo a la plancha*
Asar a la brasa	Cocinar el alimento usando brasas (no llamas)	*Puerros a la brasa*
Asar al horno	Cocinar un alimento en el horno, por la acción del calor sin mediación de ningún elemento líquido	*Merluza al horno*
Blanquear	Introducir el alimento en agua fría y calentar hasta hervir	*Blanquear las espinacas*
Cocción al vapor	Cocinar el alimento utilizando únicamente vapor de agua	*Cocer alcachofas*
Escaldar	Someter a un alimento a agua hirviendo	*Escaldar guisantes*
Estofar	Cocinar a fuego suave un alimento que ha sido previamente rehogado en agua, vino, caldo u otro líquido al que se añaden diversos condimentos	*Estofar carne*
Freír	Sumergir un alimento en una grasa (normalmente aceite) a altas temperaturas (entre 160°C y 180°C)	*Freír un huevo*
Hervir	Sumergir un alimento en un líquido (agua normalmente) hirviendo (100°C) durante un periodo determinado	*Hervir patatas*
Sofreír	Freír un alimento de manera ligera a fuego medio durante un tiempo largo.	*Sofreír cebolla*
Rebozar	Pasar un ingrediente por harina (pudiendo pasarlo por huevo) para después freírlo	*Rebozar merluza*
Empanar	Pasar un ingrediente por huevo batido y pan rallado para después freírlo	*Empanar filetes*
Saltear	Cocer un alimento con poca grasa y a fuego fuerte durante un corto tiempo removiendo constantemente	*Saltear dientes de ajo*
Rehogar	Cocer un alimento suavemente con poca grasa durante un tiempo corto	*Rehogar espárragos*
Pochar o Escalfar	Cocer un alimento en un líquido sin alcanzar la ebullición	*Pochar/escalfar huevos*
Cocción al microondas	Cocción de un alimento crudo utilizando el microondas (no nos referimos a calentar comida en el microondas, si no a su uso como técnica culinaria)	*Cocción de pollo al microondas*
Papillote	Cocción de un alimento en un envoltorio (papel de hornear/aluminio) en el que se cocina un alimento al vapor o al horno	*Lubina al papillote*
Tostar	Poner un alimento a la lumbre, para que lentamente se le introduzca el calor y se vaya desecando (sin quemarse) hasta que tome color	*Tostar pan*
Emulsionar	Mezclar líquidos que no son solubles entre sí en su forma natural	*Mayonesa*

Por favor, marque una única opción de respuesta para cada técnica culinaria

Marque el recuadro que indica la frecuencia de cada técnica empleada para cocinar **por término medio** durante el **último año**

FRECUENCIA MEDIA DURANTE EL AÑO PASADO

10. Huevo

I. HUEVO

	NUNCA O CASI NUNCA	AL MES	A LA SEMANA			AL DÍA		
		1-3	1-2	3-4	5-6	1	2	3 ó +
Asar al horno (hornear)	☐	☐	☐	☐	☐	☐	☐	☐
Al vapor	☐	☐	☐	☐	☐	☐	☐	☐
Pochar o escalfar	☐	☐	☐	☐	☐	☐	☐	☐
Hervir	☐	☐	☐	☐	☐	☐	☐	☐
Microondas	☐	☐	☐	☐	☐	☐	☐	☐
Huevo frito	☐	☐	☐	☐	☐	☐	☐	☐
Tortilla o huevo revuelto	☐	☐	☐	☐	☐	☐	☐	☐
Emulsionar (mayonesa)	☐	☐	☐	☐	☐	☐	☐	☐
Otra técnica$_1$:	☐	☐	☐	☐	☐	☐	☐	☐
Otra técnica$_2$:	☐	☐	☐	☐	☐	☐	☐	☐

11. Carnes blancas: pollo, pavo, conejo, etc…

II. CARNES BLANCAS

	NUNCA O CASI NUNCA	AL MES	A LA SEMANA			AL DÍA		
		1-3	1-2	3-4	5-6	1	2	3 ó +
A la plancha	☐	☐	☐	☐	☐	☐	☐	☐
Asar a la brasa	☐	☐	☐	☐	☐	☐	☐	☐
Asar al horno	☐	☐	☐	☐	☐	☐	☐	☐
Al vapor	☐	☐	☐	☐	☐	☐	☐	☐
Estofar	☐	☐	☐	☐	☐	☐	☐	☐
Freír	☐	☐	☐	☐	☐	☐	☐	☐
Hervir	☐	☐	☐	☐	☐	☐	☐	☐
Microondas	☐	☐	☐	☐	☐	☐	☐	☐
Rebozar o empanar	☐	☐	☐	☐	☐	☐	☐	☐
Otra técnica$_1$:	☐	☐	☐	☐	☐	☐	☐	☐
Otra técnica$_2$:	☐	☐	☐	☐	☐	☐	☐	☐

12. Carnes rojas: ternera, vaca, cerdo, toro, buey, cordero, pato, caza, etc…

III. CARNES ROJAS

	NUNCA O CASI NUNCA	AL MES	A LA SEMANA			AL DÍA		
		1-3	1-2	3-4	5-6	1	2	3 ó +
A la plancha	☐	☐	☐	☐	☐	☐	☐	☐
Asar a la brasa	☐	☐	☐	☐	☐	☐	☐	☐
Asar al horno	☐	☐	☐	☐	☐	☐	☐	☐
Al vapor	☐	☐	☐	☐	☐	☐	☐	☐
Estofar	☐	☐	☐	☐	☐	☐	☐	☐
Freír	☐	☐	☐	☐	☐	☐	☐	☐
Hervir	☐	☐	☐	☐	☐	☐	☐	☐
Microondas	☐	☐	☐	☐	☐	☐	☐	☐
Rebozar o empanar	☐	☐	☐	☐	☐	☐	☐	☐
Otra técnica$_1$:	☐	☐	☐	☐	☐	☐	☐	☐
Otra técnica$_2$:	☐	☐	☐	☐	☐	☐	☐	☐

13. Pescados y mariscos: merluza, bacalao, atún, sardina, salmón, rape, etc.

IV. PESCADOS Y MARISCOS

	NUNCA O CASI NUNCA	AL MES	A LA SEMANA			AL DÍA		
		1-3	1-2	3-4	5-6	1	2	3 ó +
Asar a la plancha	☐	☐	☐	☐	☐	☐	☐	☐
Asar a la brasa	☐	☐	☐	☐	☐	☐	☐	☐
Asar al horno	☐	☐	☐	☐	☐	☐	☐	☐
Al vapor	☐	☐	☐	☐	☐	☐	☐	☐
Estofar	☐	☐	☐	☐	☐	☐	☐	☐
Freír	☐	☐	☐	☐	☐	☐	☐	☐
Hervir	☐	☐	☐	☐	☐	☐	☐	☐
Rebozar o empanar	☐	☐	☐	☐	☐	☐	☐	☐
Microondas	☐	☐	☐	☐	☐	☐	☐	☐
Otra técnica$_1$:	☐	☐	☐	☐	☐	☐	☐	☐
Otra técnica$_2$:	☐	☐	☐	☐	☐	☐	☐	☐

14. Verduras y hortalizas: acelga, alcachofa, calabacín, calabaza, judía verde, tomate, etc…

V. VERDURAS Y HORTALIZAS

	NUNCA O CASI NUNCA	AL MES	A LA SEMANA			AL DÍA		
		1-3	1-2	3-4	5-6	1	2	3 ó +
A la plancha	☐	☐	☐	☐	☐	☐	☐	☐
Asar a la brasa	☐	☐	☐	☐	☐	☐	☐	☐
Asar al horno	☐	☐	☐	☐	☐	☐	☐	☐
Blanquear y escaldar	☐	☐	☐	☐	☐	☐	☐	☐
Al vapor	☐	☐	☐	☐	☐	☐	☐	☐
Preparación de vegetales crudos (ensalada, gazpacho)	☐	☐	☐	☐	☐	☐	☐	☐
Estofar	☐	☐	☐	☐	☐	☐	☐	☐
Freír	☐	☐	☐	☐	☐	☐	☐	☐
Hervir (verduras enteras, puré, cremas)	☐	☐	☐	☐	☐	☐	☐	☐
Microondas	☐	☐	☐	☐	☐	☐	☐	☐
Pochar o saltear	☐	☐	☐	☐	☐	☐	☐	☐
Rebozar y empanar	☐	☐	☐	☐	☐	☐	☐	☐
Otra técnica$_1$:	☐	☐	☐	☐	☐	☐	☐	☐
Otra técnica$_2$:	☐	☐	☐	☐	☐	☐	☐	☐

Por favor, marque una única opción de respuesta para cada técnica culinaria

	NUNCA O CASI NUNCA	AL MES	A LA SEMANA			AL DÍA		
		1-3	1-2	3-4	5-6	1	2	3 ó +
**15. Patata y otros tubérculos (yuca, boniato o batatata, etc…)**								
VI. TUBÉRCULOS								
Asar a la brasa	☐	☐	☐	☐	☐	☐	☐	☐
Asar al horno	☐	☐	☐	☐	☐	☐	☐	☐
Al vapor	☐	☐	☐	☐	☐	☐	☐	☐
Freír	☐	☐	☐	☐	☐	☐	☐	☐
Hervir	☐	☐	☐	☐	☐	☐	☐	☐
Microondas	☐	☐	☐	☐	☐	☐	☐	☐
Otra técnica$_1$: ______	☐	☐	☐	☐	☐	☐	☐	☐
Otra técnica$_2$: ______	☐	☐	☐	☐	☐	☐	☐	☐

	NUNCA O CASI NUNCA	AL MES	A LA SEMANA			AL DÍA		
		1-3	1-2	3-4	5-6	1	2	3 ó +
**16. Frutas: manzana, pera, plátano, melón, arándanos, piña, cereza, etc…**								
VII. FRUTA								
Asar al horno	☐	☐	☐	☐	☐	☐	☐	☐
Hervir (compota, puré)	☐	☐	☐	☐	☐	☐	☐	☐
Otra técnica$_1$: ______	☐	☐	☐	☐	☐	☐	☐	☐
Otra técnica$_2$: ______	☐	☐	☐	☐	☐	☐	☐	☐

	NUNCA O CASI NUNCA	AL MES	A LA SEMANA			AL DÍA		
		1-3	1-2	3-4	5-6	1	2	3 ó +
**17. Legumbres: garbanzos, alubias (pintas, blancas, negras), habas, guisantes, lentejas, etc…**								
VIII. LEG.								
Hervir (potajes, ensaladas, purés, hummus)	☐	☐	☐	☐	☐	☐	☐	☐
Microondas	☐	☐	☐	☐	☐	☐	☐	☐
Otra técnica$_1$: ______	☐	☐	☐	☐	☐	☐	☐	☐
Otra técnica$_2$: ______	☐	☐	☐	☐	☐	☐	☐	☐

	NUNCA O CASI NUNCA	AL MES	A LA SEMANA			AL DÍA		
		1-3	1-2	3-4	5-6	1	2	3 ó +
**18. Cereales: arroz, avena, quinoa, maíz, mijo, espelta, cebada, semillas de chía y lino, etc…**								
VII. CEREALES								
Hervir	☐	☐	☐	☐	☐	☐	☐	☐
Microondas	☐	☐	☐	☐	☐	☐	☐	☐
Tostar	☐	☐	☐	☐	☐	☐	☐	☐
Otra técnica$_1$: ______	☐	☐	☐	☐	☐	☐	☐	☐
Otra técnica$_2$: ______	☐	☐	☐	☐	☐	☐	☐	☐

Por favor, marque una única opción de respuesta para cada elaboración

**19.** Indique cuántas veces realiza los siguientes platos **elaborados en casa** *(no se consideran los comprados, precocinados o ya preparados)*	PREPARACIÓN MEDIA DURANTE EL AÑO PASADO							
	NUNCA O CASI NUNCA	AL MES	A LA SEMANA			AL DÍA		
		1-3	1-2	3-4	5-6	1	2	3 ó +
Sofrito (tomate, cebolla, ajo o puerro elaborado a fuego lento con aceite de oliva)	☐	☐	☐	☐	☐	☐	☐	☐
Salsas caseras (bechamel, tomate…)	☐	☐	☐	☐	☐	☐	☐	☐
Caldos caseros	☐	☐	☐	☐	☐	☐	☐	☐
Pizzas, cocas	☐	☐	☐	☐	☐	☐	☐	☐
Croquetas, empanadas, empanadillas, flamenquines	☐	☐	☐	☐	☐	☐	☐	☐
Pan casero	☐	☐	☐	☐	☐	☐	☐	☐
Repostería y bollería casera	☐	☐	☐	☐	☐	☐	☐	☐
Postres caseros (flan, arroz con leche…)	☐	☐	☐	☐	☐	☐	☐	☐

Por favor, marque una única opción de respuesta para cada producto

**20.** Indique cuál de estos productos utilizan en casa para cocinar y la frecuencia de su uso	USO MEDIO DURANTE EL AÑO PASADO							
	NUNCA O CASI NUNCA	AL MES	A LA SEMANA			AL DÍA		
		1-3	1-2	3-4	5-6	1	2	3 ó +
Ajo	☐	☐	☐	☐	☐	☐	☐	☐
Cebolla	☐	☐	☐	☐	☐	☐	☐	☐
Limón	☐	☐	☐	☐	☐	☐	☐	☐
Caldo precocinado comercial	☐	☐	☐	☐	☐	☐	☐	☐
Frutos secos	☐	☐	☐	☐	☐	☐	☐	☐
Mostaza	☐	☐	☐	☐	☐	☐	☐	☐
Miel	☐	☐	☐	☐	☐	☐	☐	☐
Yogur	☐	☐	☐	☐	☐	☐	☐	☐
Nata	☐	☐	☐	☐	☐	☐	☐	☐
Leche, leche evaporada	☐	☐	☐	☐	☐	☐	☐	☐
Queso	☐	☐	☐	☐	☐	☐	☐	☐
Cerveza	☐	☐	☐	☐	☐	☐	☐	☐
Vino (tinto, blanco, etc…)	☐	☐	☐	☐	☐	☐	☐	☐
Whisky u otras bebidas destiladas	☐	☐	☐	☐	☐	☐	☐	☐
Vinagre (manzana, módena…)	☐	☐	☐	☐	☐	☐	☐	☐
Otro producto$_1$: ______	☐	☐	☐	☐	☐	☐	☐	☐
Otro producto$_2$: ______	☐	☐	☐	☐	☐	☐	☐	☐

Por favor, marque una única opción de respuesta para cada grasa

**21.** Indique la **grasa** que utiliza para cocinar y la frecuencia de su uso.	PREPARACIÓN MEDIA DURANTE EL AÑO PASADO							
	NUNCA O CASI NUNCA	AL MES	A LA SEMANA			AL DÍA		
		1-3	1-2	3-4	5-6	1	2	3 ó +
Aceite de oliva virgen extra								
Aceite de oliva virgen								
Aceite de oliva								
Aceite de girasol								
Aceite de girasol alto oleico								
Mezcla de los aceites anteriores								
Manteca de cerdo								
Mantequilla								
Margarina								
Otros aceites$_1$:								
Otros aceites$_2$:								

Por favor, marque una única opción de respuesta para cada condimento y especia

Para cada **condimento y especia** marque el recuadro que indica la frecuencia de consumo **por término medio** durante el **año pasado**.	USO MEDIO DURANTE EL AÑO PASADO							
	NUNCA O CASI NUNCA	AL MES	A LA SEMANA			AL DÍA		
		1-3	1-2	3-4	5-6	1	2	3 ó +
**22. Condimentos (usados solo para cocinar, no para añadir a productos como café, yogur, etc…)**								
Sal								
Azúcar blanco								
Azúcar moreno y panela								
Edulcorante acalórico (sacarina, stevia…)								
Pastillas saborizantes								
Otro condimento$_1$:								
Otro condimento$_2$:								
**23. Especias**								
Ajo en polvo								
Albahaca								
Anís, anís estrellado								
Azafrán								
Canela								
Cilantro								
Clavo								
Comino								
Cúrcuma								
Hinojo								
Jengibre								
Laurel								
Menta, hierbabuena								
Nuez moscada								
Orégano								
Perejil								
Perejil desecado								
Pimentón, paprika								
Pimienta								
Pimienta cayena								
Romero								
Sésamo								
Tomillo								
Otra especia$_1$:								
Otra especia$_2$:								

**24.** Indique cuál de los siguientes **utensilios de cocina tiene** en su casa.	Indique si dispone de ellos	
	Sí	No
Balanza o vaso medidor de líquidos		
Freidora		
Horno		
Licuadora		
Microondas		
Olla a presión		
Parrilla (carbón o eléctrica)		
Picadora		
Robot de cocina		
Tostadora		
Túrmix o batidora electrica		
Vaporera		
Plancha		

HOME COOKING FREQUENCY QUESTIONNAIRE

The objective of this questionnaire is to know how often you cook and use different culinary techniques at home. All questions refer to the average during the last year.

1. Are you in charge of planning the weekly menus for lunch and/or dinner at home?
- ☐ Yes, I am in charge of weekly planning of lunches and/or dinners
- ☐ No, but we do plan our lunches and/or dinners weekly
- ☐ We do not plan our lunches and/or dinners weekly

2. Are you in charge or do you collaborate in the weekly household shopping? ☐ Yes ☐ No

3. Do you cook at home? ☐ Yes ☐ No ***(question 8)***

4. What percentage of homemade dishes do you cook? ☐ <25% ☐ 25-49% ☐ 50-75% ☐ >75% ☐ 100%

5. How many people do you cook for (including yourself)? ☐ 1 ☐ 2 ☐ 3 ☐ 4 ☐ 5 ☐ 6 ☐ 7 ☐ 8 or +

6. ¿Do you do batch cooking? *(batch cooking: dedicate one day to cook various meals)* ☐ Yes ☐ No

Please, choose one option for both questions

	DAYS A WEEK							HOURS A WEEK		
	1 day	2 days	3 days	4 days	5 days	6 days	7 days	<3h	3-7h	>7h
**7. How many days and hours do you spend cooking each week?** *(not including vacation: Christmas, Easter)*	☐	☐	☐	☐	☐	☐	☐	☐	☐	☐
**8. How many days and hours do you spend cooking during vacation periods (summer, Christmas, Easter)?**	☐	☐	☐	☐	☐	☐	☐	☐	☐	☐

For each one of the following questions, check the box that indicates the average frequency in the previous year.

**9. How frequently do you …**	AVERAGE FREQUENCY DURING LAST YEAR								
	NEVER OR ALMOST NEVER	MONTHLY	WEEKLY						
		1-3	1	2	3	4	5	6	7
eat homemade food for lunch (including packed lunch, at home of a relative/ friend)?	☐	☐	☐	☐	☐	☐	☐	☐	☐
eat homemade food for dinner (including packed lunch, at home of a relative/ friend)?	☐	☐	☐	☐	☐	☐	☐	☐	☐
eat lunch or dinner accompanied?	☐	☐	☐	☐	☐	☐	☐	☐	☐
eat in a restaurant or workplace cafeteria for lunch?	☐	☐	☐	☐	☐	☐	☐	☐	☐
eat in a restaurant or workplace cafeteria for dinner?	☐	☐	☐	☐	☐	☐	☐	☐	☐
buy homemade food from a restaurant or a supermarket for take-away?	☐	☐	☐	☐	☐	☐	☐	☐	☐
eat pre-cooked food for lunch (instant soups, pizzas…)?	☐	☐	☐	☐	☐	☐	☐	☐	☐
eat pre-cooked food for dinner (instant soups, pizzas…)?	☐	☐	☐	☐	☐	☐	☐	☐	☐

The following questions refer to the culinary techniques used at home for cooking. Please answer, even if you don't cook. For each food group, indicate how often each culinary technique has been used on average in the last year.
By "cooking" we mean any type of processing of fresh, frozen or blunted food. We do not consider cooking the handling of already prepared or pre-cooked foods (pizzas or lasagna, soups or creams, or rice or pasta ready to be heated in the microwave).

**DEFINITIONS**. Following, a list of the most frequent culinary techniques and their definition is displayed. They can be useful to fulfill the questionnaire.		**EXAMPLES**
Grill (griddle/pan)	To cook at a high temperature in a dry medium (a drop of oil might be added)	*Sautéing chicken*
Grill (barbecue)	To cook food using hot coals (not flame)	*Grilled leaks*
Roast (meat, vegetables), bake (pastries, fish)	To cook the food in the oven, by the action of heat without the mediation of any liquid element	*Roast the beef*<br>*Bake the hake*
Blanch	To immerse the food in cold water and heat it until boiling	*Blanch spinach*
Steam	To cook the food using only water steam	*Steaming artichokes*
Scald	To immerse the food in boiling water	*Scald the peas*
Stew	To cook the food at low heat previously sautéed in water, wine, broth or other liquid with different condiments	*Stew the meat*
Fry	To submerge the food in fat (usually oil) at high temperatures (between 160°C y 180°C)	*Frying an egg*
Boil	To submerge the food in a boiling (100°C) liquid (usually water) for a specified period of time	*Boiling potatoes*
'Sofreir'	To lightly fry the food at medium temperature during a prolonged period of time	*Stir-frying the onion*
Dredge	To coat an ingredient in flour (could also be coated with egg) in order to fry it afterwards	*Dredging hake*
Bread	To coat an ingredient with whisked egg and bread crumbs and frying it afterwards	*Breading fillets*
Stir-fry	To cook quickly in a small amount of very hot fat, constantly stirring	*Stir-fry garlic*
Sauté	To lightly cook the food with a small amount of fat during a short period of time	*Sauté asparagus*
Poach	To cook the food in a liquid without reaching boiling	*Poaching eggs*
Cook in the microwave	To cook raw foods using a microwave (we are not reffering to heating up the food in the microwave, but rather to using it as a culinary technique)	*Cooking chicken in the microwave*
'En papillote'	To cook a food in a wrap (baking paper / aluminum) in which a food is steamed or baked	*Sea bass en papillote*
Toast	To put food on the fire, so that the heat is slowly introduced and it dries (without burning) until it gains color	*Toast the bread*
Emulsify	To mix liquids that are not soluble in each other in their natural form	*Mayonnaise*

Please, only select one of the options for each culinary technique

Check the option that indicates the frequency at which each culinary technique was used on **average** in the **previous year**

AVERAGE FREQUENCY DURING THE LAST YEAR

10. Egg

I. EGGG

Technique	NEVER OR ALMOST NEVER	MONTHLY 1-3	WEEKLY 1-2	WEEKLY 3-4	WEEKLY 5-6	DAILY 1	DAILY 2	DAILY 3 or +
Roast in the oven								
Steam								
Poach								
Boil								
Microwave								
Fried egg								
Omelet or scrambled eggs								
Emulsify (mayonnaise)								
Other technique 1:								
Other technique 2:								

11. White meat: chicken, turkey, rabbit, etc.

II. WHITE MEAT

Technique	NEVER OR ALMOST NEVER	MONTHLY 1-3	WEEKLY 1-2	WEEKLY 3-4	WEEKLY 5-6	DAILY 1	DAILY 2	DAILY 3 or +
Grill (griddle/pan)								
Grill (barbecue)								
Roast in the oven								
Steam								
Stew								
Fry								
Boil								
Microwave								
Dredge or bread								
Other technique 1:								
Other technique 2:								

12. Red meat: beef, pork, bull, lamb, duck, game meat, etc.

III. RED MEAT

Technique	NEVER OR ALMOST NEVER	MONTHLY 1-3	WEEKLY 1-2	WEEKLY 3-4	WEEKLY 5-6	DAILY 1	DAILY 2	DAILY 3 or +
Grill (griddle/pan)								
Grill (barbecue)								
Roast in the oven								
Steam								
Stew								
Fry								
Boil								
Microwave								
Dredge or bread								
Other technique 1:								
Other technique 2:								

13. Fish and shellfish: hake, cod, tuna, sardine, salmon, monkfish, etc.

IV. FISH AND SHELLFISH

Technique	NEVER OR ALMOST NEVER	MONTHLY 1-3	WEEKLY 1-2	WEEKLY 3-4	WEEKLY 5-6	DAILY 1	DAILY 2	DAILY 3 or +
Grill (griddle/pan)								
Grill (barbecue)								
Bake in the oven								
Steam								
Stew								
Fry								
Boil								
Microwave								
Dredge or bread								
Other technique 1:								
Other technique 2:								

14. Vegetables: chard, artichoke, zucchini, pumpkin, green beans, tomato, etc.

V. VEGETABLES

Technique	NEVER OR ALMOST NEVER	MONTHLY 1-3	WEEKLY 1-2	WEEKLY 3-4	WEEKLY 5-6	DAILY 1	DAILY 2	DAILY 3 or +
Grill (griddle/pan)								
Grill (barbecue)								
Roast in the oven								
Blanch and scald								
Steam								
Raw vegetables (salad, cold vegetable soup)								
Stew								
Fry								
Boil (whole vegetables, puree, soup)								
Microwave								
Stir-fry								
Dredge and bread								
Other technique 1:								
Other technique 2:								

Please, only select one of the options for each culinary technique

	NEVER OR ALMOST NEVER	MONTHLY 1-3	WEEKLY 1-2	WEEKLY 3-4	WEEKLY 5-6	DAILY 1	DAILY 2	DAILY 3 or +
**15. Potato and other tubers (cassava, sweet potato, etc.)** (VI. TUBERS)								
Grill (barbecue)	☐	☐	☐	☐	☐	☐	☐	☐
Roast/bake	☐	☐	☐	☐	☐	☐	☐	☐
Steam	☐	☐	☐	☐	☐	☐	☐	☐
Fry	☐	☐	☐	☐	☐	☐	☐	☐
Boil	☐	☐	☐	☐	☐	☐	☐	☐
Microwave	☐	☐	☐	☐	☐	☐	☐	☐
Other technique1: ______________	☐	☐	☐	☐	☐	☐	☐	☐
Other technique2: ______________	☐	☐	☐	☐	☐	☐	☐	☐

	NEVER OR ALMOST NEVER	MONTHLY 1-3	WEEKLY 1-2	WEEKLY 3-4	WEEKLY 5-6	DAILY 1	DAILY 2	DAILY 3 or +
**16. Fruits: apple, pear, banana, melon, blueberries, pineapple, cherry, etc.** (VII. FRUIT)								
Roast/bake	☐	☐	☐	☐	☐	☐	☐	☐
Boil (compote, puree)	☐	☐	☐	☐	☐	☐	☐	☐
Other technique1: ______________	☐	☐	☐	☐	☐	☐	☐	☐
Other technique2: ______________	☐	☐	☐	☐	☐	☐	☐	☐

	NEVER OR ALMOST NEVER	MONTHLY 1-3	WEEKLY 1-2	WEEKLY 3-4	WEEKLY 5-6	DAILY 1	DAILY 2	DAILY 3 or +
**17. Legumes: chickpeas, beans (pinto, white, black), broad beans, peas, lentils, etc…** (VIII. LEG.)								
Boil (stews, salads, purees, hummus)	☐	☐	☐	☐	☐	☐	☐	☐
Microwave	☐	☐	☐	☐	☐	☐	☐	☐
Other technique1: ______________	☐	☐	☐	☐	☐	☐	☐	☐
Other technique2: ______________	☐	☐	☐	☐	☐	☐	☐	☐

	NEVER OR ALMOST NEVER	MONTHLY 1-3	WEEKLY 1-2	WEEKLY 3-4	WEEKLY 5-6	DAILY 1	DAILY 2	DAILY 3 or +
**18. Grains: rice, oats, quinoa, corn, millet, spelt, barley, chia and flax seeds, etc.** (VII. GRAINS)								
Boil	☐	☐	☐	☐	☐	☐	☐	☐
Microwave	☐	☐	☐	☐	☐	☐	☐	☐
Toast	☐	☐	☐	☐	☐	☐	☐	☐
Other technique1: ______________	☐	☐	☐	☐	☐	☐	☐	☐
Other technique2: ______________	☐	☐	☐	☐	☐	☐	☐	☐

Please, only select one of the options for each dish

19. Please indicate how often do you prepare the following homemade dishes *(do not take into consideration those bought, pre-cooked or already prepared)*

AVERAGE PREPARATION DURING THE LAST YEAR	NEVER OR ALMOST NEVER	MONTHLY 1-3	WEEKLY 1-2	WEEKLY 3-4	WEEKLY 5-6	DAILY 1	DAILY 2	DAILY 3 or +
Sofrito (tomato, onion, garlic or leek cooked with olive oil over low heat)	☐	☐	☐	☐	☐	☐	☐	☐
Homemade sauces (bechamel, tomato…)	☐	☐	☐	☐	☐	☐	☐	☐
Homemade broth	☐	☐	☐	☐	☐	☐	☐	☐
Pizza,	☐	☐	☐	☐	☐	☐	☐	☐
Croquettes, *empanadas* (savory pies filled with vegetables and meat or tuna (baked)) , *empanadillas* (small *empanadas* (baked or fried)), *flamenquines* (roulade of pork loin or ham, serrano ham and bacon)	☐	☐	☐	☐	☐	☐	☐	☐
Homemade bread	☐	☐	☐	☐	☐	☐	☐	☐
Homemade pastries	☐	☐	☐	☐	☐	☐	☐	☐
Homemade desserts (custard, rice pudding…)	☐	☐	☐	☐	☐	☐	☐	☐

Please, only select one of the options for each ingredient

20. Please indicate the frequency of use of each ingredient for cooking at home

AVERAGE USE DURING THE <u>LAST YEAR</u>	NEVER OR ALMOST NEVER	MONTHLY 1-3	WEEKLY 1-2	WEEKLY 3-4	WEEKLY 5-6	DAILY 1	DAILY 2	DAILY 3 or +
Garlic	☐	☐	☐	☐	☐	☐	☐	☐
Onion	☐	☐	☐	☐	☐	☐	☐	☐
Lemon	☐	☐	☐	☐	☐	☐	☐	☐
Store bought precooked broth	☐	☐	☐	☐	☐	☐	☐	☐
Nuts	☐	☐	☐	☐	☐	☐	☐	☐
Mustard	☐	☐	☐	☐	☐	☐	☐	☐
Honey	☐	☐	☐	☐	☐	☐	☐	☐
Yogurt	☐	☐	☐	☐	☐	☐	☐	☐
Whipped cream	☐	☐	☐	☐	☐	☐	☐	☐
Milk, evaporated milk	☐	☐	☐	☐	☐	☐	☐	☐
Cheese	☐	☐	☐	☐	☐	☐	☐	☐
Beer	☐	☐	☐	☐	☐	☐	☐	☐
Wine (red, white, etc…)	☐	☐	☐	☐	☐	☐	☐	☐
Whisky or other distilled beverages	☐	☐	☐	☐	☐	☐	☐	☐
Vinegar (apple, modena…)	☐	☐	☐	☐	☐	☐	☐	☐
Other product1: ______________	☐	☐	☐	☐	☐	☐	☐	☐
Other product2: ______________	☐	☐	☐	☐	☐	☐	☐	☐

Please, only select one of the options for each type of fat

**21.** Please indicate the frequency of use of each fat do you use for cooking	AVERAGE USE DURING THE LAST YEAR							
	NEVER OR ALMOST NEVER	MONTHLY	WEEKLY			DAILY		
		1-3	1-2	3-4	5-6	1	2	3 OR +
Extra virgin olive oil	☐	☐	☐	☐	☐	☐	☐	☐
Virgin olive oil	☐	☐	☐	☐	☐	☐	☐	☐
Olive oil	☐	☐	☐	☐	☐	☐	☐	☐
Sunflower oil	☐	☐	☐	☐	☐	☐	☐	☐
Sunflower oil high in oleic acid	☐	☐	☐	☐	☐	☐	☐	☐
Mix of oils mentioned above	☐	☐	☐	☐	☐	☐	☐	☐
Lard	☐	☐	☐	☐	☐	☐	☐	☐
Butter	☐	☐	☐	☐	☐	☐	☐	☐
Margarine	☐	☐	☐	☐	☐	☐	☐	☐
Other oil$_1$: ______________	☐	☐	☐	☐	☐	☐	☐	☐
Other oil$_2$: ______________	☐	☐	☐	☐	☐	☐	☐	☐

Please, only select one of the options for each seasoning and spice

For each seasoning and spice check the box that indicates the **average** frequency of consumption during the **last year.**	AVERAGE USE DURING THE LAST YEAR							
	NEVER OR ALMOST NEVER	MONTHLY	WEEKLY			DAILY		
		1-3	1-2	3-4	5-6	1	2	3 OR +
**22. Seasonings (used for cooking only, not added to products like coffee, yogurt, etc…)**								
Salt	☐	☐	☐	☐	☐	☐	☐	☐
White sugar	☐	☐	☐	☐	☐	☐	☐	☐
Brown sugar or panela	☐	☐	☐	☐	☐	☐	☐	☐
Calorie free sweeteners (saccharin, stevia…)	☐	☐	☐	☐	☐	☐	☐	☐
Flavor tablets	☐	☐	☐	☐	☐	☐	☐	☐
Other seasoning$_1$: ______________	☐	☐	☐	☐	☐	☐	☐	☐
Other seasoning$_2$: ______________	☐	☐	☐	☐	☐	☐	☐	☐
**23. Spices**								
Garlic powder	☐	☐	☐	☐	☐	☐	☐	☐
Basil	☐	☐	☐	☐	☐	☐	☐	☐
Anise, star anise	☐	☐	☐	☐	☐	☐	☐	☐
Saffron	☐	☐	☐	☐	☐	☐	☐	☐
Cinnamon	☐	☐	☐	☐	☐	☐	☐	☐
Cilantro	☐	☐	☐	☐	☐	☐	☐	☐
Clove	☐	☐	☐	☐	☐	☐	☐	☐
Cumin	☐	☐	☐	☐	☐	☐	☐	☐
Turmeric	☐	☐	☐	☐	☐	☐	☐	☐
Fennel	☐	☐	☐	☐	☐	☐	☐	☐
Ginger	☐	☐	☐	☐	☐	☐	☐	☐
Laurel	☐	☐	☐	☐	☐	☐	☐	☐
Mint, spearmint	☐	☐	☐	☐	☐	☐	☐	☐
Nutmeg	☐	☐	☐	☐	☐	☐	☐	☐
Oregano	☐	☐	☐	☐	☐	☐	☐	☐
Parsley	☐	☐	☐	☐	☐	☐	☐	☐
Dried parsley	☐	☐	☐	☐	☐	☐	☐	☐
Paprika	☐	☐	☐	☐	☐	☐	☐	☐
Pepper	☐	☐	☐	☐	☐	☐	☐	☐
Cayenne pepper	☐	☐	☐	☐	☐	☐	☐	☐
Rosemary	☐	☐	☐	☐	☐	☐	☐	☐
Sesame	☐	☐	☐	☐	☐	☐	☐	☐
Thyme	☐	☐	☐	☐	☐	☐	☐	☐
Other spice$_1$: ______________	☐	☐	☐	☐	☐	☐	☐	☐
Other spice$_2$: ______________	☐	☐	☐	☐	☐	☐	☐	☐

**24.** Please indicate whether or not you have the following kitchen gadgets.	Indicate if you have any of the following	
	Yes	No
Scale or measuring cup for liquids	☐	☐
Fryer	☐	☐
Oven	☐	☐
Blender	☐	☐
Microwave	☐	☐
Pressure cooker	☐	☐
Grill (coal or electric)	☐	☐
Mincer	☐	☐
Food processor	☐	☐
Toaster	☐	☐
Electric mixer	☐	☐
Steamer	☐	☐
Griddle	☐	☐

Supplementary Material Table S1. Characteristics of the experts group.

Number of participants	6
Sex	
Male	2
Female	4
Age (range)	37-62
Academic degree	
Chef	2
Dietitian-Nutritionist	3
Epidemiologist	1
Education level	
Graduate	3
Master	1
PhD	2

Supplementary Material Table S2. Characteristics of the population of the face validity and internal consistency study, criterion validity study and test-retest reliability and internal consistency study.

	Face validity and internal consistency	Criterion validity	Test-retest reliability and internal consistency
Number of participants	17	18	51
Sex			
Male	9	8	15
Female	8	10	36
Age (range)	23-69	43-69	22-65
Education level			
Primary	3	3	4
Secondary	5	6	15
University	9	9	32
